# Identification of Candidate Genes Associated with Meat Production of Aberdeen Angus Cattle

**DOI:** 10.3390/ani15020155

**Published:** 2025-01-09

**Authors:** Vladimir Kolpakov, Elena Bukareva, Dianna Kosyan, Alexey Ruchay, Vitaly Ryazanov

**Affiliations:** Federal Research Centre of Biological Systems and Agro-Technologies, Russian Academy of Sciences, 460000 Orenburg, Russia; vkolpakov056@yandex.ru (V.K.); elenka_rs@mail.ru (E.B.); kosyan.diana@mail.ru (D.K.); vita7456@yandex.ru (V.R.)

**Keywords:** cattle, Aberdeen Angus breed, genomic architecture, genome-wide associative studies, proactive qualities

## Abstract

The use of GWASs in agriculture allows associations between SNPs and quantitative or qualitative traits in cattle to be estimated. This study investigated the relationships among a number of economically useful carcass traits (slaughter weight, meat yield, and meat marbling) with a group of SNPs that can act as DNA markers. Blood samples from Aberdeen Angus bulls (n = 260) were used as material for SNP genotyping. Genetic architecture for the kill yield trait showed the presence of 31 SNPs. Three SNPs were found to be the most reliable: BTB-00197584 (*p* = 7.20 × 10^−5^), Hapmap46735-BTA-86653 (*p* = 5.05 × 10^−5^), and BTB-00676077 (*p* = 7.02 × 10^−5^). There were 10 SNPs associated with meat yield. For meat yield, 7 SNPs were identified, four of them exceeding the threshold of established validity: ARS-BFGL-NGS-30557 (*p* = 1.28 × 10^−12^), ARS-BFGL-NGS-68920, ARS-BFGL-NGS-30466 on chromosome 8 (*p* = 1.20 × 10^−6^ and 3.03 × 10^−6^, respectively) and ARS-BFGL-NGS-40640 (*p* = 7.10 × 10^−6^). For marbling, 11 SNPs were identified on 6 of 29 chromosomes. These SNPs can act as potential markers for productivity assessment in cattle.

## Simple Summary

This article identifies candidate genes associated with economically useful carcass traits in a population of Aberdeen Angus cattle using a GWAS. The study revealed a number of significant SNPs associated with economically useful traits (slaughter weight, meat yield, meat mar-bling). Functional annotation detected 33 genes associated with biological processes (GO BP) and 5 genes registered in the Cattle QTL database. For the *OSBPL3* and *RBFOX3* genes, within which a number of economically useful trait SNPs Hapmap39351-BTA-71190 and ARS-BFGL-NGS-11938 are localized, related with slaughter weight and meat marbling, we recommend developing tests for their inclusion in the genomic selection of beef cattle. Of interest is the *OSBPL3* gene, within which the SNPs are located, so that tests that are unique and breed-specific can be developed. Our results provide useful information for understanding the genetic architecture and its further use in breeding.

## 1. Introduction

Sequencing technologies have made it possible to study the genomes of cattle of different breeds and to analyze and compare their genetic structure [1]. The discovery of quantitative trait loci (QTL) has led to advances in the identification and comprehension of genetic modifications associated with economically significant phenotypic traits. Genome-wide association studies (GWASs), which use powerful techniques for the genetic analysis of single nucleotide polymorphism (SNP), are a relatively new approach to cattle genetic studies, which have identified different groups of candidate genes responsible for economically useful traits. This is especially important for research on cattle breeds whose genetic potential has not yet been fully realized. This is particularly true of the Aberdeen Angus breed.

The Aberdeen Angus breed of cattle is one of the most popular in the production of marbled meat. Animals of this breed are characterized by calm and malleable temperament, which makes them easy to handle and convenient for keeping on farms [2]. They also adapt easily to different housing and climatic conditions, making them suitable for different regions and farming systems. In terms of economic efficiency, the Aberdeen Angus breed has a good feed-to-meat conversion, which enables farmers to obtain higher yields at lower costs, while good fertility and high calf survival make them profitable for breeding. Earlier marker-assisted selection on Angus bulls in Hungary revealed the influence of a number of SNPs (*DGAT1*, *TG*, and *Lep*) on meat marbling [3]. There are relatively high frequencies of desirable alleles of *Lep* and *CAPN1* genes in Russian beef cattle populations and a positive effect of allele T of the Arg4Cys *LEP* polymorphism on the fatness of animals from birth to weaning [4,5]. CAPN1_316 polymorphisms were found to affect the average weight gain in Aberdeen Angus cattle [4].

Although there are studies in Europe and the US, there is very little data on the genetic structure of Aberdeen Angus cattle and their meat productivity in Russia, which makes it impossible to provide breeders with reliable information on the breed for the conservation and protection of genetic diversity and use in commercial livestock breeding.

This study identifies candidate genes associated with economically useful traits in a population of Aberdeen Angus cattle using GWASs.

## 2. Materials and Methods

### 2.1. Ethical Statement

All activities related to animal care, the collection of biological material, and phenotype characterization were carried out in exact accordance with the regulations of the Federal State Budgetary Scientific Institution “Federal Research Center for Biological Systems and Agrotechnologies of the Russian Academy of Sciences”. Copies of the minutes of the 30 April 2024, Laboratory Animal Control Commission Meeting No. 2/1, were received on 6 May 2024. Data and samples were collected, and no animals were disturbed during data collection.

### 2.2. Animals and Phenotypes

The research work was carried out on a specialized territory for fattening animals located in the Kostroma region, Russia. Young Aberdeen Angus cattle (n = 260) were selected as the object of study. The area required to accommodate one bull in an outdoor feedlot was estimated at about 10 square meters. All individuals had identical sex and age characteristics and were under the same conditions of nutrition and care. At the time of the experiment, the age of the studied individuals was 532.0 ± 1 days. After 43 days, their weight just before slaughter was 617.6 ± 1.68 kg.

Their diet consisted of a mixture of corn silage and high-moisture corn feed. The animals also had free access to wheat straw located in the center of the feedlot. The feeders were cleaned before morning feeding. The feeding and maintenance conditions were the same for the whole group. The feed contained 53.5% dry matter, 11.75% protein, 10.3% fiber, 2.35% fat, and 5.4% ash. The animals had free access to water.

At the end of the fattening period, the cattle were sent to slaughter at a commercial meat processing facility. Animals were identified with an RFID chip. Carcasses were identified with tags and placed in a refrigerator 24–48 h after slaughtering. After cooling, the carcass was cut into marketable pieces.

### 2.3. DNA Samples Collection, Genotyping and Quality Control

For the genome-wide association search, 260 blood samples were obtained for genotypic analysis. Blood samples were used to extract genomic DNA. DNA extraction and genotype determination were performed using DNA-Extran (Syntol LLC, Moscow, Russia). Genomic DNA concentration was determined using Qubit (Thermofisher, Waltham, MA, USA), while DNA abundance was measured using Nanodrop ND-1000 (Thermofisher, Waltham, MA, USA).

### 2.4. Genome-Wide Association Studies

The genotyping of animals was performed using a high-density microarray BovineSNP50 (Illumina Inc., San Diego, CA 92122, USA) containing 53 218 SNPs according to the manufacturer’s standard methodology.

Unmapped SNPs and SNPs located on X and Y chromosomes and in mitochondrial DNA were excluded. SNPs located on autosomes were investigated. Plink 1.90 [6] was used to check the quality of the genotyped breeds based on the following criteria: the call-rate for the SNPs for each individual sample is at least 90% (—mind); the call-rate for each SNP across all genotyped samples is at least 90% (—geno); the minor allele frequency (MAF) is more than 0.05 (—maf); the deviation of SNP genotypes from the Hardy-Weinberg equilibrium in the samples had a significance level of *p* < 10^−6^ (—hwe): --assoc and --linear, to realize the methodology of multiple linear regression of the trait depending on the minor allele coding (0—homozygote for the most frequent allele, 1—heterozygote, 2—homozygote for the minor allele). Thus, the final equation had the general form of the following:

Y = μ + Xb + Kw + Sc + Za + e,(1)

where *Y* is the vector of phenotypes; *μ* is the overall mean; *b* is the vector of fixed effects, including piggyback and year effects; *w* is the vector of the live weight of individuals when measurement is complete, treated as a covariance; *c* is the vector of SNP effects; *a* is the vector of random additive genetic effects with *e ~ N* (*Gσα2*), where *G* is the matrix of genomic ratios calculated from full genomic genotyping data using medium- or high-density DNA chips, and *σα2* is the variance of polygenetic additives; *K* is the regression coefficient of the live weight of individuals when the measurement is completed; *e* is the vector of residual errors with *e ~ N* (*Iσe2*), where *I* is the unit matrix and *σe2* is the residual variance; and *X*, *S,* and *Z* are the incident matrices for *b*, *c,* and *a*, respectively.

A heterozygosity test was performed to identify those samples that differed from the mean by more than three standard deviations in order to exclude them from the study. A total of 34 289 SNPs and 258 samples underwent quality control procedures and were retained for further study.

The *p* value (*p* < 1.45 × 10^−5^) of significance for single nucleotide polymorphisms was determined based on the Bonferroni correction method (0.05/*N*) [7], where *N* is the total number of SNPs remaining after quality control.

Data visualization was performed in the GWAS rMVP package using the R programming language [8].

### 2.5. Gene Identification, Function and Pathway Enrichment and Network Analysis

The region boundaries indicated according to the ARS-UCD2.0 genome assembly were applied to recognize cattle genes using the Ensembl 103 databases through the online resource [9]. The Database for Annotation, Visualization, and Integrated Discovery (DAVID), which provides researchers with a comprehensive set of functional annotation tools to understand the biological meaning of large gene lists [10], and the Cattle QTL database (https://www.animalgenome.org/cgi-bin/QTLdb/BT/index accessed on 27 May 2024), was used for functional gene annotation. Bioinformational data processing and work with graphs were carried out using R [8].

## 3. Results and Discussion

### 3.1. Information of SNPs

**Figure 1:**
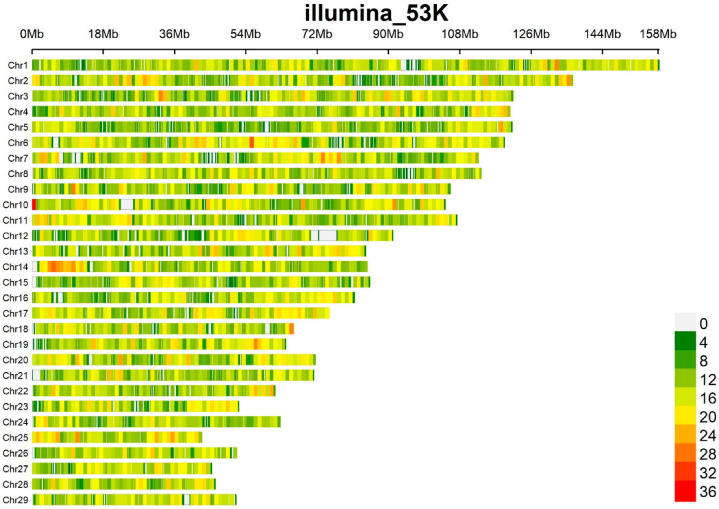
SNP density plot for the studied traits. The higher the SNP density, the redder the color.

Quality control of SNPs was performed using PLINK 1.9. After filtering, the number of SNPs was 34 289. The filtered SNPs were distributed unevenly across all 29 chromosomes (see Figure 1). The GWAS rMVP package was used to visualize SNP density across chromosomes in the R program.

The data revealed that some of the SNPs are associated with a number of phenotypic parameters that may be important economically. The data are presented in Table 1, showing the number and chromosome distribution of SNPs associated with slaughter weight, meat yield, and meat marbling.

### 3.2. Association Analysis and the Identification of Candidate Genes Related to Carcass Yield

**Figure 2:**
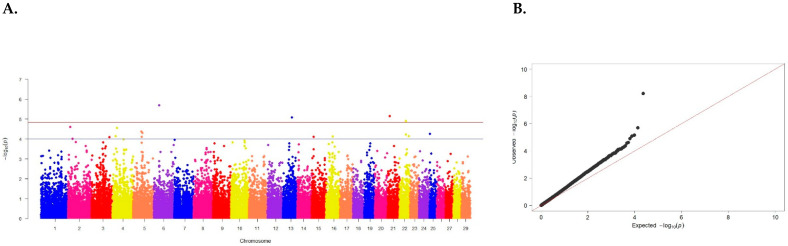
(**A**) Distribution of SNPs across cattle chromosomes in relation to confidence level (−log10 (p)) by probability summation (blue line, *p* < 0.00001) and Bonferroni criterion (red line, *p* < 1.20 × 10^−6^) for slaughter weight. (**B**) Quartile of the probability distribution of expected and observed deviations from normal distribution for confidence values.

The genetic architecture showed the presence of 31 SNPs located on 16 chromosomes but significantly associated with the trait. No SNPs exceeding the established threshold of the full genomic study were identified (Figure 2, Table 1).

Three SNPs were found to be the most reliable: BTB-00197584 on chromosome 4 (*p* = 7.20 × 10^−5^), Hapmap46735-BTA-86653 on chromosome 14 (*p* = 5.05 × 10^−5^), and BTB-00676077 on chromosome 17 (*p* = 7.02 × 10^−5^) (Table 2).

### 3.3. Association Analysis and Identification of Candidate Genes Related to Meat Yield in Cattle

**Figure 3:**
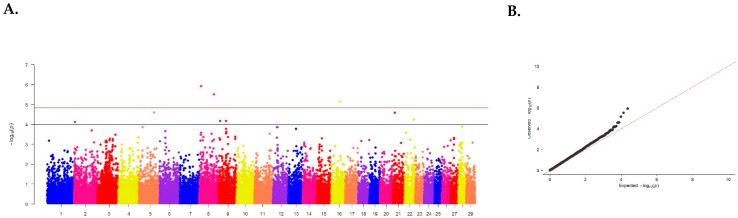
(**A**) Distribution of single nucleotide mutations on cattle chromosomes in relation to the level of confidence (−log10 (p)) by probability summation (blue line, *p* < 0.00001) and Bonferroni criterion (red line, *p* < 1.20 × 10^−6^) for meat yield. (**B**) Quartile of probability distribution of the expected and observed deviations from the normal distribution for confidence values.

Ten SNPs on chromosomes 2, 5, 8, 9, 16, and 21 are associated with the trait of meat yield (Figure 3, Table 1).

For the meat yield, 7 SNPs were identified, four of them exceeding the threshold of established reliability: ARS-BFGL-NGS-30557 on chromosome 5 (*p* = 1.28 × 10^−12^), ARS-BFGL-NGS-68920, ARS-BFGL-NGS-30466 on chromosome 8 (*p* = 1.20 × 10^−6^ and 3.03 × 10^−6^, respectively) and ARS-BFGL-NGS-40640 on chromosome 16 (*p* = 7.10 × 10^−6^) (Table 3).

### 3.4. Association Analysis and the Identification of Candidate Genes Related to Meat Marbling

**Figure 4:**
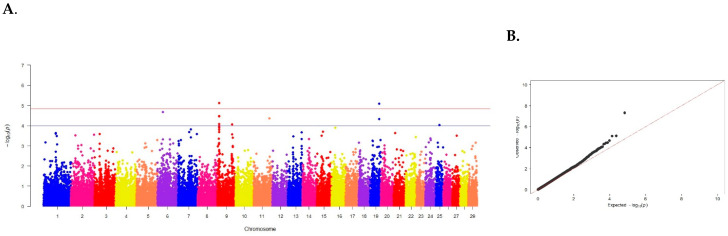
(**A**) Distribution of single nucleotide mutations on cattle chromosomes in relation to the level of confidence (−log10 (p)) by probability summation (blue line, *p* < 0.00001) and Bonferroni criterion (red line, *p* < 1.20 × 10^−6^) for meat marbling. (**B**) Quartile of probability distribution of the expected and observed deviations from normal distribution for confidence values.

The meat marbling category identified 11 SNPs on 6 of 29 chromosomes (Figure 4, Table 1).

Meat marbling—the presence of intramuscular fat—is of great economic importance for beef cattle. The Aberdeen Angus breed is considered the best, and the breed purity and origin of animals are important, as only specially bred cattle have the genetic predisposition for accumulating intramuscular fat. The genetic architecture of meat marbling and the identification of genes responsible for the formation of the best grading category by full genome association study on Russian populations of the Aberdeen Angus breed has not been previously conducted.

In our studies, 6 SNPs were identified, of which 3 exceeded the threshold of established validity: ARS-BFGL-NGS-30557 located on chromosome 5 (*p* = 4.83 × 10^−8^), BovineHD0900002742 on chromosome 9 (*p* = 7.61 × 10^−6^) and ARS-BFGL-NGS-36573 on chromosome 19 (*p* = 8.08 × 10^−6^) (see Table 4).

### 3.5. Structural Annotation of Genes Localized Within and/or in Close Proximity to Selected Regions of the Animal Genome

The structural annotation describes the exact location of various elements in the genome, such as open reading frames (ORFs), coding sequences (CDSs), exons, introns, repeats, splicing sites, regulatory motifs, start and stop codons, and promoters.

Table 5 presents the structural annotation of the genes identified for the studied traits. Structural annotation revealed the presence of 175 genes on 23 of 29 chromosomes.

### 3.6. Functional Annotation of Genes Localized Within and/or in Close Proximity to Selected Regions of the Animal Genome

Functional annotation analysis involved annotating genes with GO terms and pathway information (see Table 6).

The ***HS6ST1*** gene is associated with muscularity in Marchigiana and Chianina breeds and has significant additive associations with carcass weight in crossbred cattle slaughtered between 12 and 36 months of age with reported carcass phenotypes [11]. In our studies, this gene is correlated with meat yield by biological processes associated with alveolar development in the lungs (GO:0048286), neuronal development (GO:0048666), and labyrinthine layer blood vessel development (GO:0060716). The SNP ARS-BFGL-NGS-92825 was found within the ***HS6ST1*** gene, and therefore, it can be recommended for further test development.

The *UGGT1* gene is associated with characteristics including carcass weight and carcass fatness in Italian cattle breeds [12]. The ***OSBPL3*** gene has previously been associated with the regulation of feed intake and energy expenditure in Nellore cattle, and it acts as a lipid transporter or sensor at membrane contact sites, affecting lipid metabolism [11]. In the analysis of our bull population, this gene is correlated with slaughter weight, and the SNP Hapmap39351-BTA-71190 is located where the single nucleotide substitution occurs. The *GSDME* gene is associated with feed conversion and feed intake, and the *CFAP69* gene is associated with mammary immune system mechanisms [11].

The *DDX60* gene was previously identified as predicting buffalo semen quality and fertility [13]. The ***ELP3*** gene is significantly associated with predicted residual feed intake in commercial beef cattle littermates [14]. In our studies, the SNP ARS-BFGL-NGS-68920 was found within this gene and is correlated (*p* = 1.20 × 10^−6^) with meat yield. The *SCARA5* gene is involved in iron delivery and is located on the centromere end of chromosome 8 of the bovine genome, which is essential for the genetic predisposition of muscle mineral composition in beef cattle [15].

The BTA9:43.3–43.6 Mb region, associated with meat yield, includes the *ATG5* (autophagy 5) gene, which has an autophagy function mainly in preimplantation ovaries [16]. This autophagy function was previously found in significant amounts in ovaries under heat stress in pigs [17]. This gene has also been associated with embryo development after four- and eight-cell stages in mice [18]. An ATP-binding cassette, subfamily G (WHITE), member 5 (*ABCG5*) was identified on chromosome 11 in the 27 Mb region. *ABCG* family members are associated with cellular lipid transport into macrophages and hepatocytes in Nelore cattle [19].

The *TMEM68* gene has been linked to feed intake and growth phenotypes in cattle [20]. In our study, it was significantly associated with the slaughter weight of animals. The *TTC36* gene has been identified in cattle of the Braford breed as resistant to skin mite infestation [21].

The ***SPATA17*** gene was previously identified in beef cattle as influencing reproduction and muscle formation [22]. In this study on Aberdeen Angus cattle, it is correlated with slaughter weight, and SNP Hapmap58079-rs29010441 was detected within it.

The ***RBFOX3*** gene is a candidate regulator of mineral content in the muscle of Nelore cattle [23]. In our study, this gene is associated with meat marbling, and within it, SNP ARS-BFGL-NGS-11938 is localized on chromosome 19. The expression of the *C1QTNF1* gene was detected in the adipose tissue of lactating Holstein cows [24]. The *CLEC3B* gene is involved in calcium ion binding and encodes a protein that is important for bone mineralization, cellular response to transforming growth factor beta stimulation, the positive regulation of plasminogen activation, and the development of the body’s skeletal system [25].

The ***ABCA14*** gene is associated with the marbling category in our analysis, it was detected on chromosome 25, and within it, the SNP Hapmap43799-BTA-17809 was identified. Previously, this gene was found in a population of dairy cattle and correlated with male fertility [26]. The *ZP2* gene, discovered by DNA extraction from semen straws of Holstein bulls, is responsible for oocyst maturation at different stages [27]. The analysis of commercial frozen semen from six Holstein Friesian bulls aged 2 and 4 years shows that two genes, *SPADH1* and *SPADH2*, play a role in single fertilization [28].

By analyzing the functional annotation of the genes for the three traits studied, we can expand the understanding of the genetic architecture and select genes for further molecular validation.

**Figure 5:**
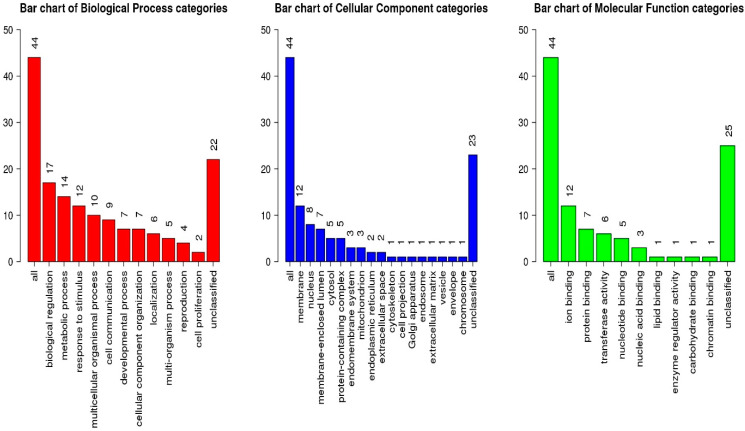
Ratio of 44 functionally identified genes across the following library groups: biological, cellular, and molecular.

Gene set enrichment analysis and gene set correlation were performed in the WebGestalt web-based program. To avoid false positives, it is necessary to designate enrichment categories using multiple tools (five enrichment tools for GO biological process, six for GO molecular function, and three for KEGG pathways) that are considered consistently enriched. Using these selection criteria, the general categories of biological, molecular, and cellular GO processes can be identified (see Figure 5).

The results showed the highest reliability (*p* = 3.91 × 10^−4^) for the GO:0007338 libraries responsible for the biological process, namely, predisposition to single fertilization (high fertility). It included the genes *SPADH1* (spermadhesin 1), *SPADH2* (spermadhesin 2), and *ZP2* (zona pellucida glycoprotein 2), and the enrichment ratio was 20,57. The following annotation of the identified genes was performed in the Cattle QTL database, and the results are presented in Table 7.

The highest number of QTLs was identified for the *RBFOX3* gene (15 QTLs). This gene and the *OSBPL3* gene, the results for which are presented in Table 6 and registered 1 QTL, are significant for the further development of tests for them and their introduction into genomic selection programs of beef cattle.

## 4. Conclusions

The results provide useful information for understanding genetic architecture and provide some foundations for molecular breeding. The overall reliability of animal genotyping was 99.5%. Functional annotation performed using the web-based program DAVID detected 33 genes associated with biological processes of the organism (GO BP) and 5 genes registered in the Cattle QTL database. For the *OSBPL3* and *RBFOX3 genes*, within which the SNPs Hapmap39351-BTA-71190 and ARS-BFGL-NGS-11938 are localized, interrelated with slaughter weight and meat marbling, we recommend developing tests for their inclusion in the genomic selection of beef cattle. Of special interest is the *OSBPL3* gene, within which the identified SNPs are located, due to which it is possible to develop unique and breed-specific tests in the future.

The study revealed a number of significant SNPs associated with economically useful traits (slaughter weight, meat yield, meat marbling) in Aberdeen Angus cattle. Genes containing SNPs (*OSBPL3*, *ABCG5*, *CRYM*, *ZP2,* and *ACADSB*) were identified mainly for their involvement in biological quality and metabolism.

## Figures and Tables

**Table 1:** Number and chromosome distribution of SNPs associated with the indicators.

Indicators	nSNP *	Chromosome Distribution
Slaughter weight, kg	31	1, 3, 4, 7, 8, 10, 11, 14, 15, 16, 17, 18, 21, 25, 26, 29
Meat yield on bone, %	10	2, 5, 8, 9, 16, 21
Marbling, cat.	11	5, 6, 9, 11, 19, 25

* nSNP is the number of reliably detected single nucleotide polymorphisms.

**Table 2:** List of SNPs significantly associated with slaughter weight.

Chromosome Number	SNP *	Position	*p*
1	ARS-BFGL-NGS-116207	36 239 709	1.75 × 10^−5^
	ARS-BFGL-NGS-103594	149 043 397	3.94 × 10^−5^
	Hapmap43997-BTA-106314	149 067 642	4.58 × 10^−5^
3	BTB-00132025	64 044 549	3.22 × 10^−5^
	BTB-00148451	106 510 754	7.05 × 10^−5^
	ARS-BFGL-NGS-27698	106 543 574	6.61 × 10^−5^
4	Hapmap39351-BTA-71190	71 031 890	9.03 × 10^−5^
	BTB-00197584	74 579 013	7.20 × 10^−5^
7	BTA-117735-no-rs	86 169 121	2.03 × 10^−5^
8	ARS-BFGL-NGS-21043	494 866	7.05 × 10^−5^
	Hapmap60592-rs29025478	79 660 034	3.18 × 10^−5^
	ARS-BFGL-NGS-2393	105 195 851	2.25 × 10^−5^
10	ARS-BFGL-NGS-87984	59 731 691	5.22 × 10^−5^
11	ARS-BFGL-NGS-6860	26 162 970	6.10 × 10^−5^
14	Hapmap40063-BTA-34263	22 983 665	8.82 × 10^−5^
	Hapmap46735-BTA-86653	25 401 722	5.05 × 10^−5^
	**ARS-BFGL-NGS-44032**	**25 425 357**	**6.69 × 10^−6^**
	UA-IFASA-8451	68 026 718	5.66 × 10^−5^
	ARS-BFGL-NGS-115921	76 472 657	2.93 × 10^−5^
	ARS-BFGL-BAC-23729	76 803 498	4.65 × 10^−5^
	ARS-BFGL-NGS-113293	77 274 386	3.78 × 10^−5^
15	ARS-BFGL-NGS-24733	29 271 879	4.01 × 10^−5^
16	Hapmap58079-rs29010441	21 212 680	8.13 × 10^−5^
17	BTB-00676077	29 057 538	7.02 × 10^−5^
18	ARS-BFGL-NGS-21074	18 772 590	4.94 × 10^−5^
21	ARS-BFGL-NGS-31918	14 915 708	5.25 × 10^−5^
	ARS-BFGL-NGS-23752	60 065 494	2.98 × 10^−5^
25	ARS-BFGL-NGS-17307	24 092 836	4.38 × 10^−5^
26	UA-IFASA-5698	42 673 967	7.93 × 10^−5^
	ARS-BFGL-NGS-32465	47 288 628	4.24 × 10^−5^
29	ARS-BFGL-NGS-9388	34 746 857	9.60 × 10^−5^

Note: *p* is the reliability of the identified SNP. * SNPs exceeding the upper threshold of reliability (*p* < 1.20 × 10^−5^) are marked in **bold**.

**Table 3:** List of SNPs significantly associated with meat yield per bone.

Chromosome Number	SNP *	Position	*p*
2	ARS-BFGL-NGS-92825	4 201 525	7.58 × 10^−5^
5	ARS-BFGL-NGS-15506	88 334 676	2.52 × 10^−5^
	**ARS-BFGL-NGS-30557**	**104 972 270**	**1.28 × 10^−12^**
8	**ARS-BFGL-NGS-68920**	**10 600 564**	**1.20 × 10^−6^**
	**ARS-BFGL-NGS-30466**	**85 786 900**	**3.03 × 10^−6^**
9	Hapmap43790-BTA-121886	8 425 284	6.59 × 10^−5^
	ARS-BFGL-NGS-170	43 855 787	6.61 × 10^−5^
16	**ARS-BFGL-NGS-40640**	**49 730 423**	**7.10 × 10^−6^**
21	ARS-BFGL-NGS-27377	14 127 028	2.59 × 10^−5^
22	ARS-BFGL-NGS-119471	53 981 070	5.79 × 10^−5^

Note: *p* is the reliability of the identified SNP. * SNPs exceeding the upper threshold of reliability (*p* < 1.20 × 10^−5^) are marked in bold.

**Table 4:** List of SNPs significantly associated with meat marbling.

Chromosome number	SNP *	Position	*p*
5	**ARS-BFGL-NGS-30557**	**104 972 270**	**4.83 × 10^−8^**
6	Hapmap42761-BTA-05111	31 442 288	2.15 × 10^−5^
9	**BovineHD0900002742**	**11 030 945**	**7.61 × 10^−6^**
	BovineHD0900002745	11 034 233	8.24 × 10^−5^
	BovineHD0900002749	11 045 602	3.50 × 10^−5^
	BTA-28590-no-rs	11 178 996	3.37 × 10^−5^
	BTA-84628-no-rs	86 231 860	8.79 × 10^−5^
11	ARS-BFGL-NGS-44412	91 578 841	4.39 × 10^−5^
19	ARS-BFGL-NGS-11938	53 239 903	4.67 × 10^−5^
	**ARS-BFGL-NGS-36573**	**53 831 725**	**8.08 × 10^−6^**
25	Hapmap43799-BTA-17809	19 123 438	9.52 × 10^−5^

Note: *p* is the reliability of the identified SNP. * SNPs exceeding the upper threshold of reliability (*p* < 1.20 × 10^−5^) are marked in **bold**.

**Table 5:** Structural annotation of genes within which and/or in close proximity to SNPs.

Item Number	Chromosome Number	SNP	Position	*p*	Sign	Gene *
1	1	ARS-BFGL-NGS-116207	36 239 709	1.75 × 10^−5^	SW	*CGGBP1*, *CSNKA2IP*
2		ARS-BFGL-NGS-103594	149 043 397	3.94 × 10^−5^		***HLCS***, *RIPPLY3*, *SIM2*
3		Hapmap43997-BTA-106314	149 067 642	4.58 × 10^−5^		
4	2	ARS-BFGL-NGS-92825	4 201 525	7.58 × 10^−5^	MY	***HS6ST1***, *UGGT1*
5						
12	3	BTB-00148451	106 510 754	7.05 × 10^−5^	SW	*MACF1*, ***BMP8A***, *KIAA0754*
13		ARS-BFGL-NGS-27698	106 543 574	6.61 × 10^−5^		
15		Hapmap39351-BTA-71190	71 031 890	9.03 × 10^−5^	SW	***OSBPL3***, *GSDME*, *PALS2*
16		BTB-00197584	74 579 013	7.20 × 10^−5^	SW	*CFAP69*, *GTPBP10*, *FAM237B*, *HUS1*, *TNS3*
20		ARS-BFGL-NGS-15506	88 334 676	2.52 × 10^−5^	MY	*CMAS*
21		ARS-BFGL-NGS-30557	104 972 270	4.83 × 10^−8^	Grade	*NTF3*
23	6	Hapmap42761-BTA-05111	31 442 288	2.15 × 10^−5^	Grade	***GRID2***
24	7	BTA-117735-no-rs	86 169 121	2.03 × 10^−5^	SW	*COX7C*
25	8	ARS-BFGL-NGS-21043	494 866	7.05 × 10^−5^	SW	*MFSD14B*, *ANXA10*, *DDX60*
26		ARS-BFGL-NGS-68920	10 600 564	1.20 × 10^−6^	MY	***ELP3***, *PNOC*, *NUGGC*, *SCARA5*
27		Hapmap60592-rs29025478	79 660 034	3.18 × 10^−5^	SW	*TUT7*
28		ARS-BFGL-NGS-30466	85 786 900	3.03 × 10^−6^	MY	***SPTLC1***, *ROR2*
29		ARS-BFGL-NGS-2393	105 195 851	2.25 × 10^−5^	SW	*PAPPA*
30	9	Hapmap43790-BTA-121886	8 425 284	6.59 × 10^−5^	MY	*ADGRB3*, *LMBRD1*
31		ARS-BFGL-NGS-170	43 855 787	6.61 × 10^−5^	MY	***PRDM1***, *ATG5*
32		BTA-84628-no-rs	86 231 860	8.79 × 10^−5^	Grade	***UST***
34		ARS-BFGL-NGS-87984	59 731 691	5.22 × 10^−5^	SW	*SPPL2A*, *USP8*
35	11	ARS-BFGL-NGS-6860	26 162 970	6.10 × 10^−5^	SW	***LRPPRC***, *DYNC2LI1*, *PLEKHH2*, *ABCG5*, *ABCG8*, *PPM1B*
37	14	Hapmap40063-BTA-34263	22 983 665	8.82 × 10^−5^	SW	*XKR4*, *TMEM68*, *TGS1*, *LYN*
38		Hapmap46735-BTA-86653	25 401 722	5.05 × 10^−5^	SW	*TOX*
39		UA-IFASA-8451	68 026 718	5.66 × 10^−5^	SW	*PTDSS1*, *MTERF3*, *UQCRB*, *GDF6*
40		ARS-BFGL-NGS-115921	76 472 657	2.93 × 10^−5^	SW	*WWP1*, *ATP6V0D2*, *CA13*, *LRRCC1*, *PSKH2*, *CA1*, *RBIS*, *CA2*, *CA3*, *E2F5*
41		ARS-BFGL-BAC-23729	76 803 498	4.65 × 10^−5^	SW	
42		ARS-BFGL-NGS-113293	77 274 386	3.78 × 10^−5^	SW	
43	15	ARS-BFGL-NGS-24733	29 271 879	4.01 × 10^−5^	SW	***PHLDB1***, *UBE4A*, *ATP5MG*, *KMT2A*, *ARCN1*, *TMEM25*, *TTC36*, *TREH*, *DDX6*
44	16	Hapmap58079-rs29010441	21 212 680	8.13 × 10^−5^	SW	***SPATA17***, *GPATCH2*
46		ARS-BFGL-NGS-40640	49 730 423	7.10 × 10^−6^	MY	***PRDM16***, *ARHGEF16*, *MEGF6*
47	17	BTB-00676077	29 057 538	7.02 × 10^−5^	SW	*SCLT1*, *JADE1*
48	18	ARS-BFGL-NGS-21074	18 772 590	4.94 × 10^−5^	SW	*TENT4B*, *ADCY7*, *BRD7*, *NKD1*
49	19	ARS-BFGL-NGS-11938	53 239 903	4.67 × 10^−5^	Grade	***RBFOX3***, *ENGASE*, *CANT1*, *LGALS3BP*, *CYTH1*, *PGS1*, *THA1*, *TMC6*, *TMC8*, *BIRC5*, *TIMP2*, *C1QTNF1*, *CEP295NL*, *USP36*, *DNAH17*, *SOCS3*, *AFMID*, *TK1*, *TMEM235*, *SYNGR2*
50		ARS-BFGL-NGS-36573	53 831 725	8.08 × 10^−6^	Grade	
52	21	ARS-BFGL-NGS-27377	14 127 028	2.59 × 10^−5^	MY	***CHD2***, *RGMA*
53		ARS-BFGL-NGS-31918	14 915 708	5.25 × 10^−5^	SW	***SLCO3A1***
54		ARS-BFGL-NGS-23752	60 065 494	2.98 × 10^−5^	SW	***CLMN***, *DICER1*
58		ARS-BFGL-NGS-119471	53 981 070	5.79 × 10^−5^	MY	*TMEM158*, *CLEC3B*
60		Hapmap43799-BTA-17809	19 123 438	9.52 × 10^−5^	Grade	***ABCA14***, *CRYM*, *ANKS4B*, *ZP2*, *LDAF1*, *DNAH3*
61	26	UA-IFASA-5698	42 673 967	7.93 × 10^−5^	SW	*DMBT1*, *SPADH2*, *SPADH1*, *FAM24A*, *PSTK*, *ACADSB*, *IKZF5*
62		ARS-BFGL-NGS-32465	47 288 628	4.24 × 10^−5^	SW	***CLRN3***, *FOXI2*, *PTPRE*
63	29	ARS-BFGL-NGS-9388	34 746 857	9.60 × 10^−5^	SW	***NTM***

Note: SW—slaughter weight; MY—meat yield; Grade—marbling category. * Genes within which the identified SNPs are localized are shown in **bold.**

**Table 6:** Functional annotation of identified genes using the web-based DAVID program.

Chromosome Number	Gene *	DAVID
1	*SIM2*	Development of the nervous system and lungs
2	***HS6ST1***	Development of lung alveoli, development of neurons, development of blood vessels of the labyrinthine layer
	*UGGT1*	Catabolic process of misfolded protein
4	***OSBPL3***	Lipid transport
	*GSDME*	Positive regulation of the immune response to tumor cells
	*CFAP69*	Positive regulation of sperm flagellar motility, reaction to odor
8	*DDX60*	Defensive reaction to the virus
	***ELP3***	Central nervous system development
	*SCARA5*	Cellular iron ion homeostasis
	***SPTLC1***	Regulation of fat cell apoptosis process
9	*ATG5*	Immune system process
11	*ABCG5*	Cholesterol efflux, cholesterol homeostasis
	*ABCG8*	Cholesterol efflux, cholesterol homeostasis, negative regulation of intestinal cholesterol absorption
14	*TMEM68*	Lipid metabolism
	*LYN*	Adaptive immune response, innate immune response
	*GDF6*	Fat cells differentiation
	*CA2*	One-carbon metabolism
	*CA1*	One-carbon metabolism
	*CA3*	One-carbon metabolism
	*CA13*	One-carbon metabolism
15	*TTC36*	Tyrosine metabolic process, development of central nervous system neurons
16	***SPATA17***	Mitotic cell cycle, spindle organization, establishment of meiotic spindle localization
18	*ADCY7*	Regulation of adaptive immune response
19	***RBFOX3***	Nervous system development
	*THA1*	Metabolic process of cellular amino acids, process of glycine biosynthesis, catabolic process of threonine
	*C1QTNF1*	Glucose metabolism regulation
22	*CLEC3B*	Ossification, positive regulation of plasminogen activation, bone mineralization
25	***ABCA14***	Lipid transport
	*CRYM*	Thyroid hormone metabolic process, thyroid hormone transport, thyroid hormone transport
	*ZP2*	Binding of spermatozoa to the zona pellucida, prevention of polyspermia
26	*SPADH2*	Single fertilization
	*SPADH1*	Single fertilization
	*ACADSB*	Fatty acid metabolism

* Genes within which the identified SNPs are localized are shown in **bold.**

**Table 7:** Functional annotation of genes registered in the Cattle QTL database.

Chromosome Number	Gene *	Cattle QTL
4	***OSBPL3***	QTL 56147
11	*ABCG5*	QTL:31769
25	*CRYM*	QTL:168108
25	*ZP2*	QTL:57176; QTL:57209; QTL:57136
26	*ACADSB*	QTL:160374

* Genes within which the identified SNPs are localized are shown in **bold**.

## Data Availability

The original contributions presented in the study are included in the article, further inquiries can be directed to the corresponding author.
